# The Effect of an Adapted Digital Mental Health Intervention for Sickle Cell Disease on Engagement: A Pilot Randomized Controlled Trial

**DOI:** 10.21203/rs.3.rs-3073103/v1

**Published:** 2023-06-28

**Authors:** Ektha Parchuri, Emily Nardo, Julia O’Brien, Lori Crosby, Jerlym Porter, Tonya Palermo, Cara E Nikolajski, Marsha Treadwell, Cheryl Hillery, Eva Szigethy, Charles Jonassaint

**Affiliations:** University of Pittsburgh School of Medicine; University of Pittsburgh; University of Pittsburgh; University of Pittsburgh; University of Pittsburgh; University of Pittsburgh; University of Pittsburgh; University of California, San Francisco; UPMC Children’s Hospital of Pittsburgh; University of Pittsburgh Medical Center; University of Pittsburgh

## Abstract

**INTRODUCTION:**

Despite promising outcomes, lack of engagement and poor adherence are barriers to treating mental health using digital CBT, particularly in minority groups. After conducting guided focus groups, a current mental health app was adapted to be more inclusive for minorities living with SCD.

**METHODS:**

Patients between the ages of 16–35 with SCD who reported experiencing anxiety or depression symptoms were eligible for this study. Once enrolled, participants were randomly assigned to receive one of two versions of a mental health app 1) the current version designed for the general population and 2) the adapted version. Baseline measures for depression, anxiety, pain and self-efficacy were completed at the start of the study and again at postintervention (minimum 4 weeks).

**RESULTS:**

Compared to baseline, pain (p = 0.03), self-efficacy (p = 0.007) and depression symptoms (p = 0.016) improved for the group that received the adapted app. Regardless of group assignment, a positive relationship (r = 0.47) was shown between app engagement and a change in depression symptoms (p = 0.042).

**DISCUSSION:**

Target enrollment for this study sought to enroll 40 participants. However, after difficulties locating qualified participants, enrollment criteria were adjusted to expand the population pool. Regardless of these efforts, the sample size for this study was still smaller than anticipated (n = 21). Additionally, irrespective of group approximately 40% of participants did not engage with the app. However, despite a small sample size and poor engagement, participants in the intervention group displayed better outcomes and showed trends for greater app interaction.

**CONCLUSION:**

These promising results should encourage future researchers to continue exploring ideal adaptations for implementing digital CBT in minority populations. Future studies should also consider implementing post-intervention surveys to help identify common factors relating to a lack of engagement.

## Introduction

African Americans living with chronic medical conditions are at high risk for depression and other mental health disorders yet are less likely to be diagnosed or receive treatment than their white counterparts (1, 2). Left untreated, depression can increase disease severity and risk for mortality (3, 4). One especially vulnerable group is patients with sickle cell disease (SCD) (5), a genetic blood disorder that primarily affects people of African descent and disproportionately impacts those living in disadvantaged circumstances (6). Sickle cell causes severe acute and chronic pain, end-organ damage, and early mortality (7).

Adolescents and young adults (AYA) with SCD, those between the ages of 18–30, are at particularly high risk for disease related complications. This development period, the transition from adolescence to adulthood, is characterized by significant biological and social changes, as well as changes in their medical care. Patients will leave their pediatric hematology team, who they have often received care from for most of their live and begin working with a new adult care team who is sometimes unfamiliar to the patient. Given the intersection of multiple significant life changes, late adolescence, and young adulthood for those with SCD is a tumultuous period, characterized by social vulnerability, increased medical complications, and high health care utilization (8, 9, 18). It is not surprising, therefore, that this age group is at high risk for mental health disorders and suicidal thoughts and behaviors (10, 11). Comorbid mental health disorders also have a deleterious impact on health behaviors and outcomes in this population. SCD patients with depressive symptoms are less likely to be adherent to therapies that prevent disease progression and are at increased risk of vaso-occlusive events (VOEs) and acute chest syndrome. Increased anxiety is associated with poorer pain-related outcomes, increased risk of pain crises, and greater opioid use. Therefore, it is of utmost importance that effective treatments are deployed to screen for and treat the mental health of AYA with SCD (35).

To address the mental health of AYA with SCD, there is a need for evidence-based mental health treatments that can be delivered in low-resource settings, are easily accessible, and are provided at a low cost. Mental health treatments delivered through mobile technology make it possible to provide high-quality, evidence-based care, at scale behavioral mental health treatment that reaches patients in under-resourced settings (12, 13). Digital cognitive behavioral therapy (CBT) is effective for treating depression and anxiety and can be easily brought to scale at low cost (14). Several meta-analyses have found digital CBT effective for treating depression and anxiety in white adults (15, 16, 17). Although, there is limited data on digital CBT in minority populations, a large-scale trial conducted in primary care clinics showed that digital CBT is equally effective for treating depression and anxiety symptoms among African American as for whites (29). Further, in a pilot randomized trial among adults with SCD, compared to usual care, digital CBT was associated with greater improvements in depressive symptoms and daily pain (37). Thus, there is early evidence to suggest digital CBT may not only work in SCD but other minority or underserved patient populations as well.

Despite the promise of digital CBT, lack of uptake and sustained engagement with digital therapies is a significant barrier to widespread use of this technology, particularly in low-resource settings serving minorities (19, 20, 21, 36). Digital CBT is consistently associated with high attrition and poor adherence (22, 23, 24). Poor uptake and adherence of digital CBT is particularly problematic in SCD where lack of engagement with the treatment can negatively affect its effectiveness (25, 26, 28). In a pilot study of digital CBT with SCD adults, although patients reported benefits from digital CBT, the average user completed only 3 of 8 possible sessions (28). Post-study interviews and a focus group revealed that while SCD patients did indeed feel the CBT skills were useful, the content was often not relevant or relatable and did not represent their real-world cultural and health experience (28). In addition, interventions with this population must address cultural stigma, a well-known barrier to recruitment and retention of racial/ethnic minorities in mental health programs. To advance the field, there is a need for innovative approaches to remediating these barriers to the provision, receipt, and benefit of digital CBT-based mental health services in low-resource settings.

Adaptation of evidence-based practices is often necessary for their successful implementation in different settings. Interventions may need to be tailored to meet the needs of the target population or address differences between the context in which the intervention was originally designed and the one into which it is delivered. We know that when digital interventions have a persuasive, user-centered design and personalized, tailored content, user involvement, adherence, and outcomes are better (12), but it is not yet clear what adaptations are critical to implementation success, and whether these adaptations can effectively address the perception that an intervention is not relatable, or the stigma of mental health treatment. Thus, an important next step for digital CBT is to determine whether adaptation can overcome challenges of uptake and engagement. Therefore, we adapted an existing digital CBT program for mental health by adding images, references and content representing SCD, chronic pain, and stressors unique to African Americans living with SCD. We then compared the adapted digital CBT to the standard program in a randomized trial of adolescent and young adults with SCD and elevated mental health symptoms.

## Methods

As part of routine clinical care, SCD patients at the University of Pittsburgh Children’s and Adult clinics completed a mental health screener. Initial eligibility for the study included SCD patients between the ages of 16–30 reporting moderate or greater symptoms of anxiety of depression (i.e., Patient Health Questionnaire [PHQ-9] or Generalized Anxiety Disorder Scale [GAD-7] > 9). Using these criteria, few patients qualified for the intervention. Eligibility criteria was adjusted to include SCD patients up to age 35 reporting mild or greater symptoms of anxiety or depression (i.e., PHQ-9 or GAD-7 > 4). Patients who met these criteria were identified as potential participants and provided with a brief explanation of the research study. Eligible patients who elected to enroll were randomized, in an equal allocation ratio, into one of two groups receiving mental health treatment via a mobile app. A total of 21 individuals living with SCD enrolled in the study. Group one (n = 11) received a mental health app designed for the general population (Control), while group two (n = 10) received an enhanced version of the same app designed to be more specific to minorities living with SCD (PLUS). Both versions of the mobile app, provided access to various CBT modules and techniques that participants could engage in at will. A group specific, asynchronous health coach was also provided via the app. Health coaches were available to answer questions and guide participants to appropriate techniques. Engagement reminders were also sent out to inactive participants at least every 3 days for up to 4 reminders. Participants were not directly informed of their group status. The study protocol was approved by the University of Pittsburgh Human Research Protections Office and was conducted in compliance with the ethical standards of the responsible institution on human subjects as well as with the Helsinki Declaration.

### Adaptations made to enhance the app for SCD

Prior to adapting the app, guided focus groups were conducted to determine ways to enhance the app to increase engagement for minorities living with SCD (38). Based on the findings of this study, and guided by the FRAME, a framework for adapting interventions (39) we identified areas of adaptation related to the content, contextual factors, and the training/education of staff delivering the intervention.

#### Images and language.

**Figure 1:**
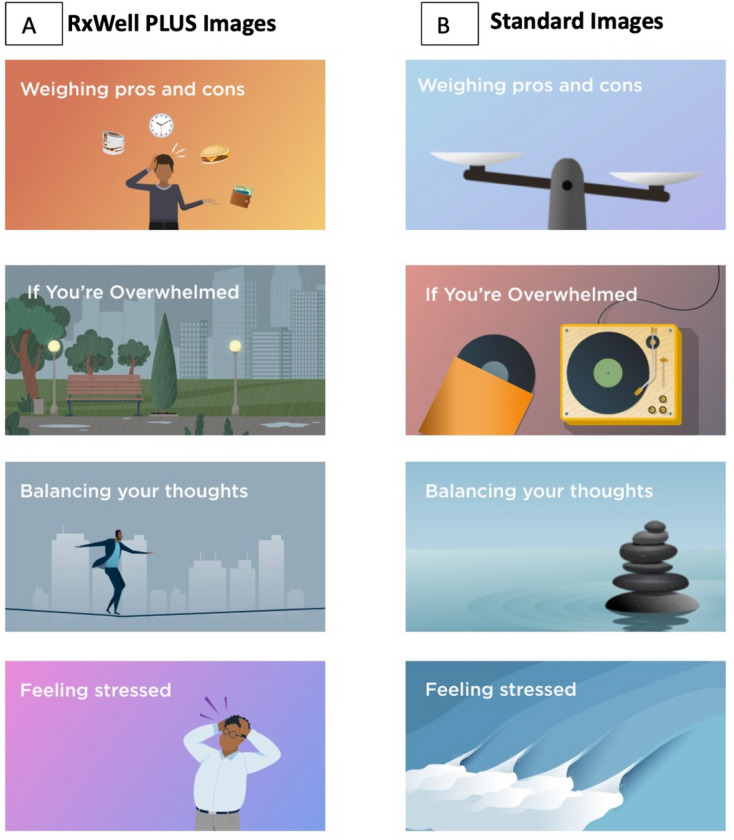
Comparison of RxWell PLUS images to Standard images in the RxWell app. (A) Four RxWell PLUS images with afrocentric and urban themes were modified from the (B) original standard images used on the RxWell app. These replacements were adapted to be more socially inclusive and relevant for the target population.

Based on these findings from our qualitative work, minor modifications were made to some of the language presented and the images. Specifically, we modified some of the images within the app to fit more Afrocentric and urban themes. Figure 1 presents four examples of how the standard CBT app images were replaced by images adapted to be more socially inclusive and relevant for the target population.

#### Introduction video.

Consistent with social cognitive theory (32), it was important for patients to know others with SCD were going through the same experience and using the same type of interventions. Consented patients randomized to the SCD adapted arm, the intervention group, received a 5-minute video that explained that mental health symptoms for AYA with SCD was common and that cognitive behavioral therapy had been shown to be effective.

#### Health Coach training and messaging.

One coach was designated to the RxWell PLUS arm who, in addition to X weeks of health coach training, also received training specific for working with adolescents and adults with SCD. The RxWell plus health coach completed an online short course on SCD that included a completion quiz and received an hour long 1:1 training with an SCD psychologist on best practices on working with individuals with SCD. The SCD-arm health coaches were also provided with 6 additional text messages specific to SCD that touched on specific wellness topics noted to by patients as important such as emotional health and nutrition. Examples of these message are:

- “Learning ways to manage stress can help lower your risk of side effects associated with SCD. What are some ways that you currently manage your stress?”
- “Did you know that your body as greater nutritional needs than those without SCD?! To help your body function and help decrease pain complications, it’s extra important to eat right. Have you ever examined your diet? Making small changes can have a BIG impact and I’m here to help!”

### Measures and Timepoints

Measures of depression [PHQ-9], anxiety [GAD-7], pain [PROMIS Pain Interference Short Form 8a], and self-efficacy [Sickle Cell Self-efficacy Scale; SCSES] were assessed at baseline (T1) and at least four weeks postintervention (T2). Paired T-tests were conducted to differences in these variables from T1 to T2. In addition, mobile app engagement was measured via the number of techniques participants used within the app, as well as the number of messages sent to health coaches between T1 and T2. Mobile app engagement was compared between groups and regression analysis was used to analyze potential relationships.

## RESULTS

The sample of enrolled patients was predominantly female, adults, who had at least some high school education or had completed high school. Baseline data and demographic characteristics were not different between Control (n = 11) and PLUS (n = 10) groups (Table 1).

Table 1 overviews demographic indicators for participants enrolled in the RxWell study in both the standard and PLUS arms. No difference in baseline characteristics of age, gender, education, and employment were noted between arms.

App engagement, as determined by the number of techniques completed within the app in addition to the number of communications with the health coach, was not different between groups. The PLUS participants accessed the app an average of 8.50 times over 4 weeks, while Control participants accessed the app an average of 5.64 times (p = 0.40). Approximately, 40% (n = 4) of PLUS and 36% (n = 4) of Control participants accessed the app 1 or fewer days throughout the 4 weeks. Regression analysis showed moderate relationship (r = 0.47) between app engagement and the score change in PHQ (p = 0.042).

In the PLUS group (n = 8), significant differences were seen between baseline and post intervention scores for pain (p = 0.03), self-efficacy (p = 0.007) and depression symptoms (p = 0.016). No differences were seen in the Control group between pre and post scores. Neither group had a significant change in anxiety or stigma from baseline to post intervention exposure (Table 2).

Table 2 compares change in PHQ-9, GAD-7, and SCSES post-intervention of both Standard and RxWell PLUS arms. Significant differences in PHQ-9 (p = 0.016) and SCSES (p = 0.007) were noted in the PLUS group, whereas no difference was noted in pre-post scores for the Standard arm. Neither group had a significant change in anxiety scores post-intervention.

## DISCUSSION

Despite high adherence to study follow-up (90.5%), approximately 40% of participants did not engage with the app outside of their initial sign-on date. Group assignment did not appear to significantly influence app engagement. However, on average, participants in the PLUS group had greater engagement. The lack of differences in engagement could in part be explained by the lower-than-expected sample size. This assumption is supported by two secondary findings from the study. First, engagement was shown to be moderately correlated with a change in PHQ scores. Consistent with prior literature, this trend suggests that participants with greater engagement showed greater improvement in depression symptoms (27). Second, improvements were seen exclusively in the PLUS group for pain, self-efficacy, and depression symptoms.

We are unable to determine whether positive changes seen within the PLUS group are attributed to the alterations made to the intervention group. While we attempted to enhance the intervention to be more appealing to minorities living with SCD, very few participants engaged with the app in a way that would have allowed them to experience the full range of augmentations. The lack of engagement seen for this study is consistent with previous studies that have shown poor adherence among this population group (26, 29). Perhaps a more innovative approach to engage participants is needed for this population. For example, participants for this study were blinded to their group during randomization. Perhaps if participants knew that they were receiving care more specific to their health condition and/or unique to their experiences as a minority, they would be more likely to explore the intervention for more than what’s required for study sign-up. Further, are ability to adapt the app design and content was limited. Initial testing with the app, participants found the app difficult to use and confusing. Improving the overall app experience may be a first step before adaptation.

Engagement may also be linked in some way to symptom severity and/or the perception of symptom severity (30, 31). For this study, an educational component regarding participant depression and anxiety scores was not directly provided. Participants may or may not have spoken with their health care providers about their scores and the meaning of those scores. It is plausible that those experiencing mild depression and/or anxiety symptoms may not have felt as though a mental health intervention was necessary compared to those with more severe symptoms. While we did not have a large enough sample to analyze this type of interaction, it is not an uncommon connection. Numerous behaviors change models recognize that the perception of severity may impact an individual’s desire or motivation to make a change (32, 33). For example, what is often referred to as the Health Belief Model acknowledges that individuals often need to feel as though their current health status is subjectively severe before choosing to act (34). Future studies may want to consider an educational component, limit recruitment to those displaying moderate to severe symptoms, or ensure to recruit a large enough sample size to explore symptom severity and its relationship to intervention engagement.

Despite poor engagement, attrition rates were much lower for this study than what has been shown in previous work. Numerous factors may contribute such as, short study length, follow-up measures that did not require additional clinic visits as well as text message reminders with a link directly to the post-intervention survey.

The sample size for this study was extremely small which did not allow for testing of covariates or group interactions. We sought to enroll an additional 20 participants, however, there was not a great enough population within the targeted clinics that qualified for participation. Future work should consider a multi-site approach to ensure a large enough population pool. In addition, our intention was to collect T2 data at four weeks post-intervention exposure, but this proved to be difficult with our present sample. Future studies should prioritize adherence to the follow-up schedule, so that differences between patient engagement are more comparable.

## CONCLUSION

Nevertheless, digital CBT holds promise as a widely available, effective treatment for mental health among AYA with SCD. Given the severe burden untreated depression and anxiety places on this group of patients, who already face significant health challenges, it is imperative that these treatments are adapted and implemented in order to meet the needs of young SCD patients. While tailoring may not be critical to promote uptake of digital CBT, this study shows preliminary evidence that tailoring is a critical part of adapting such interventions to patients with SCD.

## Figures and Tables

**Table 1:** Baseline Demographic Characteristics of Enrolled Participants

Enrolled in study	Standard (n = 11)	RxWell PLUS (n = 10)	P-value
**Age (mean)**	22.73	20.70	0.464
SD	6.0	6.43	
Min-Max	16–35	16–34	
**Gender**			>0.999
Male	36.4%	30%	
Female	63.6%	70%	
**Education**			
Some High School	36.4%	40%	
High School	27.3%	40%	
Some College	27.3%	10%	
College	9.09%	0%	
Graduate	0%	10%	
**Employment**			
Yes	54.5%	30%	
No	45.5%	60%	
Other	0%	10%	

**Table 2:** Comparison of PHQ-9, GAD-7, and SCSES outcomes between Standard and PLUS arms.

	Standard (n = 11)				RxWell PLUS(n = 10)				Comparison
Variables	Pre	Post	Diff	P value	Pre	Post	Diff	P Value	P Value
**PHQ-9**	11.18	10.09	1.091	.523	12.50	6.75	5.750	.016	.078
**GAD-7**	9.27	8.91	.364	.789	8.43	3.14	5.286		.170
**SCSES**	29.70	31.30	−1.600	.345	28.29	32.14	−3.857	.007	.298
